# Associations of Rs3744841 and Rs3744843 Polymorphisms in Endothelial Lipase Gene with Risk of Coronary Artery Disease and Lipid Levels in a Chinese Population

**DOI:** 10.1371/journal.pone.0162727

**Published:** 2016-09-09

**Authors:** Gaojun Cai, Bifeng Zhang, Chunyan Ma, Ganwei Shi, Weijin Weng, Sheliang Xue

**Affiliations:** 1 Department of Cardiology, Wujin hospital affiliated to Jiangsu University, Changzhou, Jiangsu Province, China; 2 Department of Pathology and Molecular Medicine, McMaster University, Ontario, Canada; Sudbury Regional Hospital, CANADA

## Abstract

**Objective:**

The aim of the present study was to assess the association between the 2037T/C and 2237G/A polymorphisms in the *EL* gene and the risk of CAD and lipid levels in a Chinese population.

**Methods:**

A case-control study including 706 patients with CAD and 315 controls was performed. The polymerase chain reaction-restriction fragment length polymorphism (PCR-RFLP) method was used to identify the genotypes.

**Results:**

The *EL* 2037 T/C polymorphism was associated with CAD risk and HDL-C levels. No significant differences were found between the *EL* 2237 G/A genotypes and CAD risk and lipid levels in the whole population. However, carriers of the 2237 A allele had higher Apo A1 levels than those with the 2237 GG genotype and in the CAD subgroup (*P* = 0.044). The CAD cases have a significantly lower frequency of the C-G haplotypes than the controls, and the T-A haplotype was significantly more common in the CAD patients than in the controls.

**Conclusions:**

Our study concluded that the *EL* 2037 T/C polymorphism was associated with CAD risk and HDL-C levels, and that the C allele might be a protective factor against CAD in the Chinese Han population. In addition, the *EL* 2237 A allele might be associated with an increased Apo A1 level in CAD subjects.

## Introduction

Coronary artery disease (CAD) is one of the main, clinical manifestations of atherosclerosis. Epidemiology data shows that, until 2030, CAD will remain a major cause of human death [1]. Environmental and genetic factors affect the occurrence and development of CAD. In addition to the well-known risk factors, such as age, smoking, high blood pressure, diabetes, hyperlipidemia, etc., studies have found that a variety of gene polymorphisms are independent risk factors of CAD, such as *PCSK9*, and *APOA5* [2, 3].

Endothelial lipase (EL), which belongs to the lipase family, is composed of 483 amino acids and its molecular weight is about 55 KD [4]. The *EL* gene is localized on chromosome 18q21.1. Research shows that EL is synthesized not only by endothelial cells, but also by other types of cells, such as macrophages and hepatocytes. According to the previous studies, the substrate for EL is high density lipoprotein cholesterol (HDL-C). EL is the key enzyme to regulate HDL-C levels, and hydrolyzes HDL-C, to generate free fatty acids and low-fat Apo A1. The results of large clinical studies have showed that serum EL concentrations were negatively correlated to the HDL-C level but positively associated with CAD risk [5, 6].

In 2002, deLemos identified 17 single nucleotide polymorphisms in the *EL* gene for the first time [7]. In recent years, several studies on the 584 C/T (rs2000813) and -384 A/C (rs3813082) polymorphisms have been carried out [8–10]. However, the associations between these two polymorphisms and lipid levels and CAD risk were inconsistent, which might be related to factors such as race and sample size. Compared to the 584 C/T and -384 A/C polymorphisms, the 2037 C/T (rs3744843) and 2237 G/A (rs3744841) polymorphisms have been studied less. These two polymorphisms exist in exon 10 of the *EL* gene. In 2003, Yamakawa-Kobayashi *et al*. [11] studied the relationship between several *EL* gene polymorphisms (including 2037 T/C and 2237 G/A) and lipid levels in 340 healthy Japanese school-aged children. They found that the HDL-C level in subjects with the 2237 GG genotype was 56.6± 10.4 mg/dl, which was significantly higher than in the other genotypes (GA: 52.6± 10.0 mg/dl and AA: 52.3± 11.3 mg/dl, *P* = 0.011). However, they did not find that the 2037C/T polymorphism was associated with lipid levels.

In addition, the associations between the *EL* 2037 T/C and 2237 G/A polymorphisms and the risk of CAD have never been studied before. Therefore, we conducted the present study to evaluate the relationships between these two polymorphisms and CAD risk and lipid levels in a Chinese Han population.

## Methods

### Study subjects

A total of 1021 unrelated Chinese Han subjects, were enrolled in this hospital-based case-control study. Of the 1021 subjects, 706 were CAD patients (507 males and 199 females, mean age of 63.72±10.03 years) and 315 were healthy control subjects (142 males and 173 females, mean age of 58.85±9.50 years). All subjects were randomly selected between February 2009 and May 2015 from the Department of Cardiology of Wujin Hospital, which is affiliated to Jiangsu University. Diagnosis of CAD was confirmed according to the World Health Organization (WHO) criteria from 1978 and/or >50% luminal stenosis in at least one major coronary vessel by angiography. Of the 1021 individuals, 976 subjects underwent coronary angiography (CAG) examination. The CAG was performed using the radial artery. The major coronary vessels include the left main, left anterior descending, left circumflex, right coronary artery, and large branches (diameter of vessel is more than 2.0 mm). The results were evaluated by two experienced cardiologists. Forty five patients with acute myocardial infarction (AMI) did not undergo CAG examination. Diagnoses of these subjects followed the WHO criteria: (1) a clinical history of ischemic type chest pain or chest distress (at least 30 minutes); (2) typical changes in electrocardiography (ECG); and, (3) dynamic changes in myocardial enzyme levels for cTnT or cTnI. According to the CAG results, 295 patients had single-vessel disease (SVD) and 325 patients had multi-vessel disease (MVD).

Fifteen patients were not assessed for lipid profiles and were excluded when we analyzed the associations between gene polymorphisms and lipid levels.

The controls, who were selected from individuals admitted to the hospital to rule out CAD, were diagnosed with a luminal stenosis of <50% of the major coronary arteries and without typical chest pain. The diagnosis of essential hypertension (EH) was in accordance with features described in our previous report [12]. Subjects with advanced liver or kidney failure, malignant tumors, asthma, endocrine disease, major surgery or trauma within one month, peripheral artery disease, family history and medical history of heart disease, were excluded. The study was approved by the Ethics Committee of Wujin hospital and conducted according to the Declaration of Helsinki. All subjects participating in this study gave their written informed consent.

### Collection of basic information

A special form was designed to record the basic information for participating individuals, including age, gender, history of EH and DM, smoking, history of statin use, results of CAG and coronary computed tomography angiography (CTA).

### Biochemical analysis

Total cholesterol (TC), triglyceride (TG), HDL-C and LDL-C were detected by enzymatic methods. Apo A1, Apo B and LP(a) were detected by radio-immunoassay methods. All of the biochemical indexes were analyzed by automatic biochemical analyzer (Olympus AU5400).

### DNA genotyping

Genomic DNA was isolated from peripheral blood leukocytes using standard phenol-chloroform extraction methods and was stored at -70°C. Genotypes were determined by using the polymerase chain reaction-restriction fragment length polymorphism (PCR-RFLP) method. The amplification of the rs3744843 and rs374841 gene polymorphisms proceeded using the same primers. A total volume of 40ul of reaction mix included; 1μL of genomic DNA, 1μL of forward primer (forward: 5'-GTT ACT GCT GAG GAC CCA C-3'), 1μL of reverse primer (5'-TAG AAA TCC CAA CTC CAC TG-3'), 20μL of GoTaq^®^ Green Master Mix 2× and 17ul of deionized water. Amplification reaction conditions were: denaturation for 5 min at 94°C, followed by 35 cycles at 94°C for 15 s, 58°C for 30 s, 72°C for 30s, and then incubation at 72°C for 10 min. The PCR product was digested with restriction enzymes (*Sma I* for rs3744843, *BseD I* for rs3744841) at 55°C overnight. Then, the digested PCR product was separated by 1.5% agarose gel electrophoresis. Some of the PCR products were genotyped by sequencing to confirm the results (Genewiz, Suzhou, China).

### Statistical analysis

The Statistical Package for the Social Sciences 17.0 (SPSS Inc., Chicago, IL, USA) was used for all statistical analysis. The sample size and power were assessed by the Power and Sample size Calculations [13]. Hardy-Weinberg equilibrium (HWE) was examined by the χ^2^ goodness-of-fit test. Continuous variables are expressed as means ± standard deviation (SD) and compared using independent-sample *t*-tests. The normality of the distributions was assessed using the Kolmogorov-Smirnov test, and if necessary, a Mann-Whitney test was used to assess the skewed variables. Comparison among multiple groups was done by one-way analysis of variance (one-way ANOVA). Qualitative variables including genotype and allele frequencies were assessed by the χ^2^ test. Multivariate logistic regression analysis was performed to evaluate the independent impact of *EL* SNPs on the risk of CAD, and was adjusted for traditional risk factors, including age, gender, EH, DM, smoking, dyslipidemia. SHEsis online software was used to assess the linkage disequilibrium analysis and haplotype analysis. A *P* value <0.05 (2-tailed) was considered statistically significant.

## Results

### Clinical characteristics of subjects

Table 1 shows the clinical characteristics of included individuals. The age of CAD group was 63.72± 10.03 years, which was higher than that of the control group (58.85± 9.50 years, *P*< 0.001). The percentages of males, smoking status, EH and DM in the CAD group were also significantly higher than in the control group (*P*< 0.001). Compared with the control group, the CAD patients had significantly higher TG, LDL-C and Apo B levels (*P*< 0.05). On the contrary, HDL-C and Apo A1 levels in the CAD group were significantly lower than in the control group. The levels of TC and LP (a) in the CAD group were higher than in the control group, but the difference was not significant.

**Table 1:** Clinical characteristics between CAD and control groups.

Characteristics	CAD(n = 706)	Controls(n = 315)	*P*
Male [n(%)]	507 (71.81%)	142 (45.08%)	**<0.001**
Age (years)	63.72 ± 10.03	58.85 ± 9.50	**<0.001**
EH [n(%)]	487 (68.98%)	180 (57.14%)	**<0.001**
DM [n(%)]	185 (26.20%)	31 (9.84%)	**<0.001**
Smoking [n(%)]	231 (32.72%)	61 (19.37%)	**<0.001**
Statin use [n(%)]	57 (8.07%)	6 (1.90%)	**<0.001**
TC (mmol/l)	4.55± 1.00	4.50± 0.94	0.407
TG (mmol/l)	1.87± 1.33	1.77± 1.27	**0.021**
HDL-C (mmol/l)	1.07± 0.27	1.20± 0.35	**<0.001**
LDL-C (mmol/l)	2.80± 0.87	2.58± 0.70	**<0.001**
Apo A1 (g/L)	1.20± 0.26	1.26± 0.24	**<0.001**
Apo B (g/L)	0.94± 0.28	0.89± 0.29	**0.009**
Lp (a) (g/L)	0.24± 0.27	0.22± 0.29	0.309
CAG [n(%)]	669 (94.76%)	307 (97.46%)	0.052

CAD, coronary artery disease; EH, essential hypertension; DM, diabetes mellitus; TC, total cholesterol; TG, triacylglycerols; HDL-C, high density lipoprotein cholesterol; LDL-C, low density lipoprotein cholesterol; CAG, coronary angiogram; Apo, apolipoprotein.

### *EL* 2037 T/C polymorphism

The genotypic and allelic distributions of the *EL* 2037T/C are presented in Table 2. The genotypic frequencies were in accordance with HWE in both controls and cases. The frequencies of TT, TC and CC genotypes were 62.89%, 33.14%, and 3.97% in the CAD group, compared with 53.02%, 41.27%, and 5.71% in the control group, respectively. We grouped TC and CC genotypes together as carriers of the C allele, because the CC genotype was minor. There was a significant difference in the TT genotype and the TC+CC genotype between CAD and control groups. Compared with TT genotype, the frequency of TC+CC genotype was significantly lower in the CAD group (*P* = 0.004, OR = 0.666, 95%CI = 0.509–0.871). The non-pooled results are presented in S1 Table Compared with the T allele, the frequency of the C allele was lower in the CAD group than in the control group (20.54% *vs* 26.35%, *P* = 0.004, OR = 0.722, 95%CI = 0.580–0.900).

**Table 2:** The genotypic and allelic distribution of *EL* gene polymorphisms in CAD and control subjects.

SNPs	CAD [n(%)]	Controls [n(%)]	*X*^2^	*P*
**2037 T/C (rs3744843)**				
TT	444 (62.89%)	167 (53.02%)	9.060	**0.011**
TC	234 (33.14%)	130 (41.27%)		
CC	28 (3.97%)	18 (5.71%)		
T	1122 (79.46%)	464 (73.65%)	8.481	**0.004**
C	290 (20.54%)	166 (26.35%)		
**2237 G/A (rs3744841)**				
GG	255 (36.12%)	138 (43.81%)	5.632	0.060
GA	349 (49.43%)	134 (42.54%)		
AA	102 (14.45%)	43 (13.65%)		
G	859 (60.84%)	410 (65.08%)	3.335	0.075
A	553 (39.16%)	220 (34.92%)		

SNPs, single nucleotide polymorphisms; CAD, coronary artery disease; *P* values were calculated by *X*^2^ test.

Logistic regression analysis, with adjustment for the traditional CAD risk, including sex, age, current smoker status, EH, DM and lipid levels, showed that the TC+CC genotype of the *EL* 2037 T/C polymorphism remains significantly associated with decreased CAD risk (*P* = 0.018, adjusted OR = 0.736, 95%CI = 0.570–0.950).

In the whole population, we found that the HDL-C level was significantly different among the different *EL* 2037 C/T genotypes (*P* = 0.030). After pairwise comparison, we found the individuals with the CC genotype had higher HDL-C levels than those with the TT genotype (*P* = 0.029) (S2 Table). The subjects with the TC+CC genotype had an increased serum HDL-C level as compared with the TT genotype carriers (TC+CC: 1.14± 0.33 mmol/l versus TT: 1.09± 0.29 mmol/l, *P* = 0.023). The non-pooled results are presented in S2 Table. In the subgroup analysis stratified by phenotype of disease, gender and age, the significant association of the *EL* 2037T/C polymorphism with HDL-C only existed in the control subgroup (*P* = 0.016) (Table 3).

**Table 3:** Effect of *EL* 2037T/C genotypes on serum lipid levels (TT *vs*. TC+CC).

Genotypes	TC (mmol/L)	TG (mmol/L)	HDL-C (mmol/L)	LDL-C (mmol/L)	Apo A1 (g/L)	ApoB (g/L)	LP(a) (g/L)
**All** ^**#**^							
TT (n = 602)	4.51± 0.98	1.82± 1.22	1.09± 0.29	2.73± 0.82	1.22± 0.26	0.92± 0.27	0.23± 0.27
TC+CC (n = 404)	4.56± 0.98	1.87± 1.43	1.14± 0.33	2.74± 0.83	1.22± 0.25	0.93± 0.31	0.24± 0.30
*P*	0.794	0.831	**0.023**	0.884	0.748	0.736	0.452
**CAD subgroup**							
TT (n = 435)	4.54± 1.01	1.80± 1.19	1.07± 0.27	2.81± 0.86	1.21± 0.27	0.93± 0.27	0.23± 0.26
TC+CC (n = 257)	4.55± 0.97	1.99± 1.54	1.07± 0.29	2.80± 0.89	1.19± 0.23	0.95± 0.30	0.26± 0.29
*P*	0.666	0.244	0.846	0.896	0.349	0.529	0.123
**Control subgroup**							
TT (n = 167)	4.44± 0.89	1.86± 1.31	1.16± 0.32	2.53± 0.68	1.24± 0.21	0.89± 0.25	0.23± 0.27
TC+CC (n = 147)	4.58± 0.99	1.66± 1.21	1.25± 0.37	2.62± 0.72	1.28± 0.26	0.89± 0.32	0.21± 0.31
*P*	0.177	0.103	**0.016**	0.195	0.139	0.914	0.500
**Male subgroup**							
TT (n = 396)	4.42± 1.00	1.81± 1.34	1.06± 0.29	2.70± 0.88	1.17± 0.23	0.91± 0.28	0.22± 0.26
TC+CC (n = 262)	4.41± 0.92	1.82± 1.34	1.08± 0.32	2.68± 0.82	1.20± 0.28	0.90± 0.28	0.23± 0.26
*P*	0.497	0.643	0.481	0.794	0.191	0.909	0.572
**Female subgroup**							
TT (n = 206)	4.69± 0.92	1.89± 1.20	1.16± 0.28	2.78± 0.76	1.27± 0.22	0.94± 0.26	0.24± 0.28
TC+CC (n = 142)	4.85± 1.02	1.87± 1.35	1.20± 0.31	2.87± 0.82	1.28± 0.24	0.97± 0.32	0.28± 0.36
*P*	0.450	0.304	0.218	0.287	0.759	0.366	0.302
**< 60 years subgroup**							
TT (n = 230)	4.56± 1.01	2.02± 1.51	1.09± 0.29	2.72± 0.85	1.22± 0.22	0.93± 0.27	0.23± 0.28
TG+GG (n = 147)	4.49± 0.89	1.93± 1.51	1.15± 0.33	2.66± 0.73	1.23± 0.26	0.91± 0.33	0.21± 0.28
*P*	0.499	0.253	0.071	0.484	0.643	0.629	0.523
**≥ 60 years subgroup**							
TT (n = 372)	4.48± 0.97	1.69± 0.99	1.10± 0.28	2.74± 0.81	1.21± 0.28	0.91± 0.26	0.23± 0.25
TG+GG (n = 257)	4.61± 1.02	1.84± 1.39	1.13± 0.33	2.78± 0.88	1.21± 0.25	0.93± 0.30	0.26± 0.31
*P*	0.286	0.491	0.139	0.501	0.933	0.402	0.156

#: Patients absent of lipid profiles was excluded.

As shown in Table 4, there was no significant effect of the 2037T/C polymorphism on the angiographic severity of CAD (*P* = 0.457).

**Table 4:** Effect of EL 2037 T/C genotypes and allele on angiographic severity of CAD.

	Genotypes			Alleles	
	TT (n)	TC (n)	CC (n)	T	C
**SVD (n = 295)**	182	97	16	461	129
**MVD (n = 325)**	203	111	11	517	133
***X***^***2***^		1.566		0.365	
***P***		0.457		0.578	

SVD, single-vessel disease; MVD, multi-vessel disease.

### *EL* 2237 G/A polymorphism

The genotype frequencies of *EL* 2237 G/A were also compatible with HWE in both the controls and the cases. The distributions of the *EL* 2237 G/A genotype and allele frequencies are presented in Table 2. The frequencies of GG, GA and AA genotypes were 36.12%, 49.43%, and 14.45% in the CAD patients, and 43.81%, 42.54%, and 13.65% in the controls, respectively. The frequency of the A allele was 39.16% in the CAD patients and 34.92% in the control subjects. No significant differences in the genotype (*P* = 0.060) or allele (*P* = 0.075) frequencies were found between the CAD group and the control group.

When the GA and AA genotypes were combined as the A allele, no significant differences were found between the 2237G/A polymorphisms and any lipid levels in the whole population (Table 5). However, in the subgroup analysis, based on the phenotype of disease, the 2237 A allele carriers had higher Apo A1 levels than the GG homozygote individuals in the CAD subgroup (*P* = 0.044).

**Table 5:** Effect of *EL* 2237 G/A genotypes on serum lipid levels.

Genotypes	TC (mmol/L)	TG (mmol/L)	HDL-C (mmol/L)	LDL-C (mmol/L)	Apo A1 (g/L)	ApoB (g/L)	LP(a) (g/L)
**All**							
GG (n = 390)	4.54±1.01	1.86±1.36	1.11±0.29	2.73±0.84	1.21±0.24	0.93±0.29	0.24±0.29
GA+AA (n = 616)	4.53±0.96	1.82±1.28	1.12±0.31	2.73±0.82	1.22±0.27	0.92±0.28	0.24±0.27
*P*	0.154	0.781	0.636	0.991	0.247	0.678	0.940
**CAD subgroup**							
GG (n = 252)	4.54±1.00	1.93±1.50	1.05±0.26	2.81±0.89	1.17±0.23	0.95±0.29	0.24±0.28
GA+AA (n = 440)	4.55±1.00	1.83±1.23	1.08±0.28	2.80±0.86	1.21±0.28	0.93±0.28	0.24±0.27
*P*	0.582	0.828	0.228	0.876	**0.044**	0.572	0.961
**Control subgroup**							
GG (n = 138))	4.54±1.04	1.72±1.07	1.20±0.32	2.59±0.72	1.27±0.24	0.89±0.31	0.23±0.31
GA+AA (n = 176)	4.47±0.86	1.801.40	1.20±0.37	2.57±0.69	1.25±0.24	0.88±0.27	0.22±0.27
*P*	0.541	0. 752	0.952	0.725	0.624	0.794	0.854
**Male subgroup**							
GG (n = 251)	4.41±1.00	1.81±1.34	1.06±0.29	2.70±0.88	1.17±0.23	0.91±0.28	0.22±0.26
GA+AA (n = 407)	4.42±0.95	1.82±1.34	1.08±0.32	2.68±0.82	1.20±0.28	0.91±0.28	0.22±0.26
*P*	0.955	0.910	0.481	0.794	0.191	0.909	0.572
**Female subgroup**							
GG (n = 139)	4.77±1.00	1.95±1.41	1.18±0.29	2.80±0.76	1.27±0.22	0.96±0.32	0.27±0.34
GA+AA (n = 209)	4.74±0.94	1.84±1.58	1.18±0.30	2.83±0.81	1.28±0.23	0.95±0.27	0.24±0.30
*P*	0.364	0.714	0.983	0.661	0.785	0.636	0.472
**< 60 years subgroup**							
GG (n = 155)	4.56±0.95	1.86±1.26	1.11±0.28	2.71±0.78	1.20±0.22	0.92±0.28	0.21±0.25
GA+AA (n = 222)	4.51±0.98	2.07±1.66	1.11±0.33	2.68±0.82	1.24±0.24	0.93±0.31	0.24±0.30
*P*	0.633	0.578	0.885	0.722	0.169	0.680	0.300
**≥ 60 years subgroup**							
GG (n = 235)	4.53±1.05	1.86±1.43	1.10±0.30	2.75±0.87	1.21±0.24	0.93±0.30	0.26±0.31
GA+AA (n = 394)	4.53±0.95	1.69±0.98	1.12±0.30	2.76±0.82	1.22±0.28	0.91±0.26	0.24±0.26
*P*	0.924	0.487	0.474	0.839	0.629	0.380	0.332

As shown in Table 6, there was no significant effect of the 2237 G/A polymorphism on the angiographic severity of CAD (*P* = 0.675).

**Table 6:** Effect of *EL* 2237 G/A genotypes and allele on angiographic severity of CAD.

	Genotypes			Alleles	
	GG (n)	GA (n)	AA (n)	G (n)	A (n)
**SVD (n = 295)**	109	149	37	367	223
**MVD (n = 325)**	114	160	51	388	262
***X***^**2**^	1.282			0.819	
***P***	0.527			0.382	

SVD, single-vessel disease; MVD, multi-vessel disease.

### Linkage disequilibrium and haplotype analysis

There was a significant linkage disequilibrium between the 2037 T/C and 2237 G/A SNPs (|D’| = 0.941). The four haplotypes (C-A, T-A, C-G and T-G) of these two SNPs are listed in Table 7. Compared with the C-A haplotype, the frequency of the C-G haplotype was significantly lower in the CAD cases than in the controls (*P* = 0.013, OR = 0.755, 95%CI = 0.605–0.943), and the T-A haplotype was significantly higher in the CAD patients than in the control subjects in the Chinese Han population (*P* = 0.031, OR = 1.242, 95%CI = 1.020–1.513).

**Table 7:** Haplotypes analysis in CAD and control subjects.

Haplotypes	2037 T/C	2237 G/A	Case (freq)	Control (freq)	*P*	OR (95%CI)
H1	C	A	1.78 (0.001)	8.22 (0.013)	-	-
H2	T	A	551.22 (0.390)	211.78 (0.336)	0.031	1.242 (1.020–1.513)
H3	C	G	288.22 (0.204)	157.78 (0.250)	0.013	0.755 (0.605–0.943)
H4	T	G	570.78 (0.404)	252.22 (0.400)	0.970	0.996 (0.822–1.207)

freq, frequency; OR, Odds ratio; CI, Confidence Interval.

## Discussion

To our best knowledge, this is the first study to evaluate the relationship between the *EL* 2037 T/C and 2237 G/A polymorphisms and CAD risk and lipid levels in a Chinese population. Our data showed that the 2037 C allele is independently and significantly associated with the reduced risk of CAD. In contrast, the 2237 G/A polymorphism showed no significant association with CAD.

It is generally recognized that CAD is a multifactorial disease [14]. The occurrence and development of CAD are influenced by environmental and genetic factors. Dyslipidemia is a risk factor for CAD [15]. High LDL-C and/or low HDL-C levels can contribute to the process of atherosclerosis, and eventually lead to the occurrence of CAD. In the present study, the CAD patients had significantly higher TG, LDL-C and Apo B levels compared with the control group. On the contrary, HDL-C and Apo A1 levels in the CAD group were significantly lower than in the control group. Sixty three individuals received statin treatment before they were enrolled in this study. This might influence the results. But when the 63 individuals were excluded from our analysis, the results did not change significantly.

Recent research advances have indicated that gene polymorphisms (including SNPs and haplotypes) are associated with dyslipidemia and CAD [16]. *EL* gene polymorphisms, first reported by deLemos *et al*. in 2002, might be associated with the risk of CAD and serum lipid levels. But the results were inconsistent. In 2003, Yamakawa-Kobayashi *et al*.'s study, evaluated the relationship between the *EL* gene polymorphisms (2037 T/C and 2237 G/A) and lipid levels in Japanese school children and showed that the 2237G/A polymorphism was associated with HDL-C levels. Although variants in the *EL* gene have also been genotyped in much larger Genome-wide association studies of lipid levels and CAD, most of the studies were performed on patients of European ancestry [17, 18].

The present study showed that the *EL* 2037 T/C and 2237 G/A polymorphisms existed in the Chinese Han population, and that the genotypic distributions of these two polymorphisms are in accordance with the HWE. In the whole population, the frequencies of TT, TC and CC genotypes were 59.84%, 35.65%, and 4.51%, respectively. The frequency of CC genotype was significantly lower in Chinese Han population than in Japanese population (4.51% *vs* 7.64%, *P* = 0.035). The C allele frequency was 22.33%, which was not different from that reported for the Japanese school children (22.33% *vs* 25%) [11]. The frequencies of TT, TC and CC genotypes were 62.89%, 33.14%, and 3.97% in the CAD group, compared with 53.02%, 41.27%, and 5.71% in the control group, respectively. Genotype frequencies between these two groups are significantly different (*P* = 0.011). The C allele frequency was markedly lower in the CAD group than in the controls (20.54% *vs* 26.35%, *P* = 0.004). The C allele might be a protective factor for CAD in the Chinese Han population. In our study, we also evaluated the association of the *EL* 2037T/C polymorphism with lipid levels. In the whole population, the carriers with the TC+CC genotypes had higher HDL-C levels than those with the TT genotype (*P* = 0.023). This polymorphism was in the 3’ untranslated region of exon 10. The actual mechanism is not clear. We speculate that this variant may reduce the synthesis of EL or weaken the function of EL in HDL-C metabolism, which may increase the HDL-C levels and reduce the CAD risk. In the next step, we will evaluate the association between this variant and the expression of EL.

In the subgroup analysis, the significant association of the *EL* 2037T/C polymorphism with HDL-C only existed in controls (*P* = 0.016). We hypothesized that the variant may reduce the synthesis of EL, decrease the degradation of HDL-C, and/or protect patients against the CAD risk. Logistic regression analysis with adjustment for the traditional CAD risk showed that the TC+CC genotype of *EL* 2037 T/C remains a protective factor for CAD (*P* = 0.018, adjusted OR = 0.736, 95%CI = 0.570–0.950).

In our study, we found that the genotype and allele frequencies of 2237 G/A were not significantly different between the Chinese population and the Japanese population [11]. In the CAD patients, the frequencies of 2237 G/A genotypes GG, GA, and AA were 36.12%, 49.43%, and 14.45%, compared with 43.81%, 42.54%, and 14.45% in the controls, respectively. There was no significant difference in the genotype frequency of the 2237G/A polymorphism between the cases and the controls (*P* = 0.060). The 2237 A allele frequency was 39.16% in the CAD subjects and 34.92% in the controls. The distribution of the 2237 A allele was not significantly different between the cases and the controls (*P* = 0.075). In addition, we found that the 2237G/A polymorphism was not significantly associated with any lipid levels in the whole population. However, in the stratified analysis, the carriers with the 2237 A allele had higher Apo A1 levels than those with the 2237 GG genotype in the CAD subgroup (*P* = 0.044). Although the Chinese CAD patients with the 2237 AA genotype had higher Apo A1 levels, which was different from Japanese school children, the association between 2237G/A polymorphism and CAD in Chinese population was not reach the statistic significance. The genotype-CAD association might also is affected by environmental factors.

Our results were different from those reported by Yamakawa-Kobayashi *et al*. [11] Yamakawa-Kobayashi *et al*. concluded that *EL* 2037 T/C polymorphism was not associated with lipid levels, whereas individuals with *EL* 2237 GA/AA genotype had significant lower HDL-C levels than those with GG genotype. The discrepancy might lie in the racial difference in the study subjects. The individuals in our study were adults admitted to our hospital for chest pain, while the population enrolled in Yamakawa-Kobayashi *et al*.’s study were school-age Japanese children.

In our study, the severity of CAD was measured by the number of diseased vessels, with more than 50% luminal stenosis. Based on the number of affected coronary vessels, the CAD patients were divided into SVD and MVD groups. The results revealed that neither the 2037 T/C nor the 2237 G/A polymorphism was associated with the severity of CAD.

Haplotype analysis may provide more genetic information than analysis of one single locus [19]. Our study is the first haplotype-based case-control study to evaluate the association between the human *EL* gene and CAD in the Chinese Han population. We demonstrated that the C-G haplotype was a protective factor for CAD (*P* = 0.013, OR = 0.755, 95%CI = 0.605–0.943), whereas the T-A haplotype might be a risk factor for CAD (*P* = 0.031, OR = 1.242, 95%CI = 1.020–1.513). We speculated that the subjects with the T-A haplotype might have lower HDL-C levels and have higher CAD risk than those with other haplotypes.

Several limitations of the current study should be considered. Firstly, the sample size is relatively small, especially the control group. The possibility of false positives cannot be completely ruled out. However, according to Dupont and Plummer, the power in this study was 0.807. When the power is set as 0.8, the sample size of cases is at least 694 (there were 706 cases in our study). Additional studies with larger sample size are important for Chinese populations and other ethnicities. Secondly, the age and sex distributions between CAD and control groups were not matched in our study, which may affect the interpretation of the result. Thirdly, some individual subjects used statins, which might affect the results although exclusion of these individuals did not change the resulst. Finally, case-control studies have advantages in identifying disease-related genes, as well as disadvantages in detecting gene-environment interactions [20]. Therefore, prospective studies are necessary in the future.

In conclusion, our data showed that the *EL* 2037 T/C polymorphism was associated with CAD risk and HDL-C levels, and the C allele might be a protective factor against CAD in the Chinese Han population. In addition, individuals with the *EL* 2237 A allele had higher Apo A1 levels than those with the 2237 GG genotype in CAD subjects. Further studies are needed to elucidate the underlying mechanisms of our findings.

## Supporting Information

S1 Data (XLS)

S1 Table The genotypic distribution of *EL* 2037 T/C polymorphism in CAD and control subjects. (DOC)

S2 Table Effect of *EL* 2037T/C genotypes on serum lipid levels. (DOC)
