# Supplementary material for: Associations of Rs3744841 and Rs3744843 Polymorphisms in Endothelial Lipase Gene with Risk of Coronary Artery Disease and Lipid Levels in a Chinese Population
Source: PLoS One. 2016 Sep 9;11(9):e0162727. doi: 10.1371/journal.pone.0162727 (PMC5017691; doi:10.1371/journal.pone.0162727)
Supplement: S1 Table — (DOC) [file pone.0162727.s002.doc]

**Supplement table 1. The genotypic distribution of *EL* 2037 T/C polymorphism in CAD and control subjects**

| **Genotypes** | **Controls** | **CAD** | **OR (95% CI)** | ***P*** |
| --- | --- | --- | --- | --- |
| TT (n=611) | 167 | 444 | 1.00 (Ref.) |  |
| TC (n=364) | 130 | 234 | 0.765 (0.634-0.924) | 0.006 |
| CC (n=46) | 18 | 28 | 0.698 (0.476-1.024) | 0.091 |

OR, odds ratio; CI, confidence interval.
