# Supplementary material for: Associations of Rs3744841 and Rs3744843 Polymorphisms in Endothelial Lipase Gene with Risk of Coronary Artery Disease and Lipid Levels in a Chinese Population
Source: PLoS One. 2016 Sep 9;11(9):e0162727. doi: 10.1371/journal.pone.0162727 (PMC5017691; doi:10.1371/journal.pone.0162727)
Supplement: S2 Table — (DOC) [file pone.0162727.s003.doc]

**Supplement table 2. Effect of *EL* 2037T/C genotypes on serum lipid levels**

| **Genotypes** | **TC (mmol/l)** | **TG (mmol/l)** | **HDL-C (mmol/l)** | **LDL-C (mmol/l)** | **Apo A1 (g/l)** | **Apo B (g/l)** | **Lp (a) (g/l)** |
| --- | --- | --- | --- | --- | --- | --- | --- |
| TT (n=602) | 4.51± 0.98 | 1.82± 1.22 | 1.09± 0.29 | 2.73± 0.82 | 1.22± 0.26 | 0.92± 0.27 | 0.23± 0.27 |
| TC (n=358) | 4.57± 0.98 | 1.91± 1.48 | 1.13± 0.33 | 2.74± 0.84 | 1.22± 0.25 | 0.93± 0.31 | 0.24± 0.27 |
| CC (n=46) | 4.48± 0.97 | 1.57± 0.92 | 1.20± 0.36 | 2.68± 0.77 | 1.21± 0.21 | 0.86± 0.27 | 0.30± 0.44 |
| F | 0.535 | 1.584 | 3.525 | 0.136 | 0.122 | 1.356 | 1.317 |
| *P* | 0.586 | 0.206 | **0.030** | 0.873 | 0.885 | 0.258 | 0.269 |
